# Do Interventions with Diet or Dietary Supplements Reduce the Disease Activity Score in Rheumatoid Arthritis? A Systematic Review of Randomized Controlled Trials

**DOI:** 10.3390/nu12102991

**Published:** 2020-09-29

**Authors:** Josefine Nelson, Helen Sjöblom, Inger Gjertsson, Stine M. Ulven, Helen M. Lindqvist, Linnea Bärebring

**Affiliations:** 1 The Department of Biosciences and Nutrition, Stockholm University, 17177 Stockholm, Sweden; fysioterapi@perita.se; 2 Biomedical Library, Gothenburg University Library, University of Gothenburg, 40530 Gothenburg, Sweden; helen.sjoblom@ub.gu.se; 3 Department of Rheumatology and Inflammation Research, Institute of Medicine, Sahlgrenska Academy, University of Gothenburg, 40530 Gothenburg, Sweden; inger.gjertsson@rheuma.gu.se; 4 Department of Nutrition, Institute of Basic Medical Sciences, University of Oslo, Blindern, 0317 Oslo, Norway; smulven@medisin.uio.no; 5 Department of Internal Medicine and Clinical Nutrition, University of Gothenburg, 40530 Gothenburg, Sweden; helen.lindqvist@gu.se

**Keywords:** Rheumatoid Arthritis, DAS28, diet

## Abstract

The aim was to compile the evidence from Randomized Controlled Trials (RCTs) of diet or dietary supplements used to reduce disease activity in adults with Rheumatoid Arthritis (RA). Searches were performed in the databases PubMed, Scopus and Cochrane. Only RCT studies of diets, foods or dietary supplements, looking at effects on the Disease Activity Score in 28 joints (DAS28) among adults with RA, published in peer-reviewed journals, were included. A total of 27 articles were included—three of whole diets (Mediterranean diet, raw food and anti-inflammatory diet), five of food items, five of n-3 fatty acids, five of single micronutrient supplements, four of single antioxidant supplements and five of pre-, pro- or synbiotics. Studies that showed moderate strength evidence for positive effects on disease activity in RA included interventions with a Mediterranean diet, spices (ginger powder, cinnamon powder, saffron), antioxidants (quercetin and ubiquinone), and probiotics containing Lactobacillus Casei. Other diets or supplements had either no effects or low to very low strength of evidence. In conclusion, RCT studies on diet or dietary supplements are limited in patients with RA, but based on the results in this review there is evidence that some interventions might have positive effects on DAS28.

## 1. Introduction

Rheumatoid arthritis (RA) is a chronic autoimmune disease characterized by systemic inflammation and joint destruction. RA affects 0.5–1% of the western population and many patients have an active disease, despite pharmacological treatment [1]. Persistent disease activity causes the destruction of bone and cartilage, and negatively impacts quality of life due to pain, fatigue, depression and decreased function [2]. Many patients with RA report that their dietary intake affects their disease symptoms [3], and request dietary advice to complement their existing treatment [4]. However, there is a lack of evidence for dietary effects on disease activity in RA, and consequently no such dietary guidelines exist.

Several nutrients and dietary components interact with the immune system and could affect disease activity in RA. These include omega-3 fatty acids, antioxidants [5], vitamin D [6], vitamin B6 [7], zinc and selenium [8]. In addition, pre-, pro- and synbiotics could have positive effects on RA [9,10]. The Mediterranean diet or Healthy Nordic Diet combine intakes of dietary fiber, antioxidants and omega-3 fatty acids through a high intake of fruit, berries, vegetable, whole grains, legumes, vegetable oil and fish [11,12], and could therefore be beneficial in RA. Other diets that could be beneficial are diets that are high in prebiotics in the form of dietary fiber, such as raw food [13] or vegan diets [14].

Despite indications that diet and dietary components could have positive effects on RA, few studies have summarized the results from randomized controlled trials (RCTs) in a systematic manner. A Cochrane review from 2009 [15] compiled the evidence for dietary interventions in RA, but excluded studies on dietary supplements. In addition, new studies have been published in the last decade, warranting an updated survey of the field. There is currently no fully encompassing, contemporary review that includes both diet and dietary supplements and their effect on disease activity in RA. The aim of this systematic review is therefore to compile and rate the evidence from RCTs of diet or dietary supplements used for reducing disease activity, measured by the commonly used composite instrument Disease Activity Score in 28 joints (DAS28) in adults with RA.

## 2. Materials and Methods

**Figure 1:**
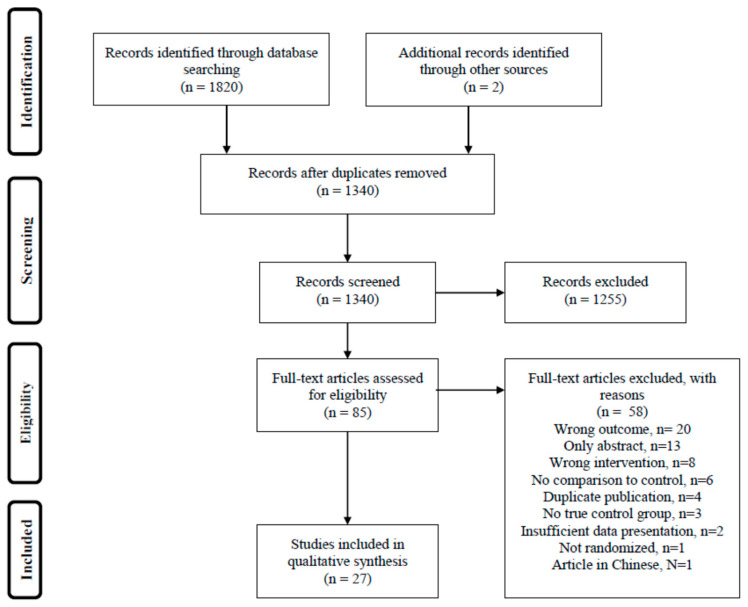
Flow chart of the article selection process according to the Preferred Reporting Items for Systematic Reviews and Meta-analyses (PRISMA).

This systematic review was conducted and reported according to the Preferred Reporting Items for Systematic Review and Meta-Analyses Protocols (PRISMA) format [16] (Figure 1). The study protocol was registered in the International Prospective Register of Systematic Reviews (PROSPERO, no CRD42019141014). A Population, Intervention, Comparison and Outcomes (PICO) statement was used to define the research question *“Can intervention with diet, food or dietary supplements reduce disease activity in RA?”* (Table S1).

The inclusion criteria for this systematic review were articles that studied the following:

- Adults (≥18 years) with RA;
- Randomized controlled trials—either parallel or crossover designs;
- Both or either diet or dietary supplements, including whole diet, specific foods, spices, herbs, nutrients, dietary antioxidants and pre-, pro- or synbiotics;
- Disease activity measured by DAS28 or DAS (see below) as an outcome of the intervention.

There were no limitations on study durations, dropout rates, participant gender or nutritional status. Dietary supplements were defined as formulations containing micronutrients, amino acids or fatty acids.

The exclusion criteria for this systematic review were as follows:

- Animal models;
- Observational studies;
- Non-randomized trials;
- Trials that lack a control group or regimen, including those comparing high dose to low dose;
- Results that were not peer reviewed, such as conference abstracts or publications in journals with no peer review process;
- Studies on natural remedies of a traditional medicinal type or herbal remedies, or food extracts;
- Studies that include other measures of disease activity than DAS/DAS28;
- Studies of other types of rheumatic diseases, including juvenile idiopathic arthritis.

For the clinical assessment of disease activity in RA, the most commonly used instrument is DAS28. DAS28 is a composite score that includes the status of 28 joints (tenderness and swelling), inflammation markers’ erythrocyte sedimentation rate (ESR) or C-reactive protein (CRP) in combination with self-perceived health using a visual analogue scale (VAS). A DAS28 score <2.6 indicates remission, 2.6–3.2 low disease activity, 3.2–5.1 intermediate disease activity and >5.1 high disease activity [17].

### Data Collection and Processing

The detailed search strategy is presented in Table S2. Searches (24 June 2019 and 21 March 2020) in the databases PubMed, Scopus and Cochrane library were performed by a trained librarian from the Biomedical Library at the University of Gothenburg, and delivered to the assessors in Rayyan. Two blinded assessors screened the abstracts, and articles that seemed to meet the criteria for inclusion were read in full length. Data were extracted using a pre-specified protocol. If data were presented from both intention to treat (ITT) and per-protocol analyses, both results were extracted but ITT was considered the primary analysis. If data on both DAS28-ESR and DAS28-CRP were presented, both results were extracted but DAS28-ESR was considered the main outcome variable.

The included articles were assessed in terms of quality using the Agency for Healthcare Research and Quality assessment tool for clinical trials according to the checklist by the Nordic Nutrition Recommendations 5 working group [18]. The individual studies were graded as A (considered high-quality with low risk of bias and likely valid results), B (moderate quality, with some risk of bias) or C (low-quality with significant risk of bias that may invalidate the results). After quality review, the studies were grouped by intervention type and a summary of evidence was given to each group, using GRADE for RCT studies [19]. According to GRADE, the evidence was graded as very low (+), low (++), moderate (+++) or high (++++). Each intervention type was assessed for problems with bias, consistency in results between trials, relevance, precision and risk of publication bias. No meta-analysis was attempted as the study designs and results were expected to be too heterogeneous.

## 3. Results

The searches resulted in a total of 1340 individual articles. Two additional articles were identified from references used by other articles. After screening the abstracts, 85 articles were read in full length, and 27 met the inclusion criteria and were included. The remaining 58 studies were excluded based on wrong outcome, abstract only, insufficient data presentation, no comparison to control group, dual publishing, wrong intervention, no true control group, not randomized or language other than English or Scandinavian (Figure 1). Summaries of the 27 included studies are shown in Table 1, Table 2 and Table 3. Quality of evidence is summarized in Table 4.

### 3.1. Studies on Whole Diets, Foods or Meals

Three RCT studies investigated the effects of whole diets (Mediterranean diet, uncooked vegan diet, and Anti-inflammatory Mediterranean style diet) on DAS28; two parallel trials [20,21] and one crossover trial [22]. All studies were performed in the Nordic region (Sweden and Finland), but were performed and written by different researchers. Intervention diets were provided, in part or in full, to the participants in all three studies, but only the study by Vadell et al. provided the same amount of foods to the control group. The remaining two studies provided either no food [21] or food in the initial phase [20] to the control group. In two of the studies, the participants in the control group were instructed to follow their habitual diet, while in the study by Vadell et al., the participants received a control diet that was based on the national average intake. The number of participants randomized in the studies varied from 43 to 56. Both genders were included but women were in the majority. At study baseline, disease activity was intermediate for all three studies. Sköldstam et al. found a significant improvement in DAS28 (effects on tender joints and CRP) when comparing the Mediterranean diet intervention to control diet (habitual diet) after 12 weeks. Vadell et al. found tendencies towards positive effects after the 10 week Anti-inflammatory Mediterranean style diet intervention, but these were not significant in adjusted comparisons to the control diet [22]. Nenonen et al. found no effect on DAS28 from the 12 week raw food vegan diet intervention [21]. The quality of the studies was high for the study by Vadell et al., intermediate for the study by Sköldstam et al., and low for the study by Nenonen et al. In sum, the quality of evidence that a Mediterranean style diet reduced DAS28 in RA was considered moderate. The quality of evidence for effects of vegan, raw food diet was very low and too limited to draw any conclusions.

Two RCT studies investigated the effects of food items on disease activity—one parallel trial studying the intake of cranberry juice [23] and one crossover trial studying the intake of blue mussels [24]. The studies included 39 and 41 participants, all female. Lindqvist et al. compared the effect of one daily ready-to-eat meal (5 days/week) for 11 weeks, supplemented with either 75 g blue mussels or chicken/red meat [24]. Thimoteo et al. compared a usual diet supplemented with 500 mL/day low calorie cranberry juice to a usual diet, for 3 months [23]. The baseline disease activity was intermediate in both studies. Thimoteo et al. found no significant effect of DAS28. However, Lindqvist et al. found a decrease in DAS28 in ITT analysis (effects on general health), but not in per protocol analysis. The quality of both studies was intermediate. The quality of evidence that a daily consumption of blue mussels reduces DAS28 in RA was considered low. Cranberry juice does not seem to reduce DAS28 in RA (moderate quality of evidence).

### 3.2. Studies on Spices in High Doses

Three studies investigated the effects of spices as capsules or tablets on DAS28. All three studies were conducted in Iran, by different research groups. The studies included 40–70 participants; all women, except for one study [25] that included 11% men. Aryaeian et al. compared 1.5 g/day ginger power in capsules to placebo capsules, in a three month parallel RCT [25]. Shishehbor et al. studied 2 g/day cinnamon power (Cinnamomum burmannii) in comparison to placebo capsules in a two month RCT [26]. Hamidi et al. compared 100 mg/day of saffron to placebo in a 3 month RCT [27]. All three studies showed significant improvement in DAS28 in comparison to the placebo. Shishehbor et al. and Hamidi et al. both found effects on swollen joints, and the former also found an effect on CRP (Aryaeian et al. did not report results on the individual components of DAS28). The studies were of high [26] and intermediate quality [25,27]. The quality of evidence was considered moderate that daily large doses of cinnamon, ginger, and saffron reduces DAS28 in RA.

### 3.3. Studies on n-3 Fatty Acid Supplements

Five studies investigated the effects of fatty acid supplements for 2.5–4 months on disease activity in RA. The studies included predominately women. The interventions were heterogeneous and the n-3 fatty acids were delivered in different vehicles. Galaragga et al. provided 97 participants with either placebo or capsules with 10 g/day cod liver oil for 12 weeks [28]. Das Gupta gave their 100 participants 3 mg/d of n3-fatty acids in capsules in addition to drug Indomethiacin, while controls received only the drug for 12 weeks [29]. In 2018, Dawcynski et al. provided their 38 participants with either foods enriched with 2.36 mg/d of n3-fatty acids from a micro algae, or with conventional foods for 10 weeks [30]. In 2009, Dawcynski et al. compared 2.4 g/day of n3-fatty acids in the form of enriched dairy products to commercial dairy products in 45 patients with RA [31]. Remans et al. gave their 66 participants either a liquid nutritional supplement containing 1.7 g/d of n3 PUFA (EPA, DHA, DPA and ALA), other macro- and micronutrients (specified) or a placebo drink [32]. At baseline, disease activity was intermediate or high. Only one [29] of the studies showed a significant effect on DAS28 in comparison to control (effects on swollen and tender joints). The study quality was intermediate for all studies. Overall, the studies showed no effect of 1.7–3 g/d of n-3 fatty acids on DAS28 (low quality evidence).

### 3.4. Studies on Vitamin D Supplements

Two studies investigated the effect of vitamin D supplementation on disease activity in RA, in comparison to placebo. The studies included 22–150 participants, predominately women. The study duration was 3 months in one study [33], and 12 months in the other [34]. In both studies, very large doses of vitamin D were given, and the effect was compared to placebo in the control group. Buondonno et al. used a single dose of 7500 µg of vitamin D3 (calculated as average per day of follow up, 83 µg/day) [33]. Hansen et al. intervened with three weekly doses of 1250 µg/week of vitamin D2 (corresponding to 180 µg/day), followed by two monthly doses of 1250 µg (corresponding to 83 µg/day) for month 2–11. Both the intervention group and the control group received 1500 mg/d of calcium [34]. The baseline disease activity was low in the study by Hansen et al. and high in the study by Buondonno et al. None of the studies found an effect of vitamin D on DAS28, compared to control. Study quality was intermediate [33] and low [34]. Overall, the studies showed no effect of vitamin D supplementation on DAS28 (low quality evidence).

### 3.5. Studies on Other Micronutrients

Rastmanesh et al. compared 6 g/day of potassium in the form of enriched grape juice to conventional grape juice among 38 hypokalemic women with RA in a parallel study design [35]. The baseline disease activity was high. After one month, there was a significant reduction in DAS28 in the intervention group compared to control (effects on tender and swollen joints, CRP, ESR and general health). The quality of the study was considered intermediate. The strength of evidence that potassium in the form of enriched juice reduces DAS28 in hypokalemic women with RA was considered low.

Shishavan et al. randomized 64 female participants with RA in remission either 10 µg/day of vitamin K1 as a chewable tablet, or placebo [36]. After two months, there was no significant difference in DAS28 between the groups. The study was considered to be of high quality. The strength of evidence was considered low that vitamin K reduce DAS28 in RA.

Van Ede et al. compared 1 mg/day folic acid or 2.5 mg/week of folinic acid, in addition to Methotrexate, to placebo among 434 women (71%) and men [37]. All three groups received Methotrexate treatment, and folinic or folic acid were given in order to study the effects on efficacy and toxicity. At baseline, disease activity was intermediate. Neither folic nor folinic acid had any effect on DAS28. The study was considered to be of intermediate quality. Based on this study, folic or folinic acid do not reduce DAS28 in RA (moderate quality of evidence).

### 3.6. Studies on Single Antioxidants

Four studies from Iran or Egypt, from different research groups, investigated the effects of single antioxidants on disease activity in RA; in three of the studies the effect was compared to placebo [38,39,40] and in one the effect was compared to regular treatment [41]. The antioxidant interventions lasted two–three months and comprised of 1200 mg/d alpha lipolic acid [38], 500 mg/d quercetin [39], 1 g/d resveratrol [41] and 100 mg/d ubiquinone [40]. There were 50–100 participants per study, predominately women.

Gargari found no effect on DAS28 of alpha lipolic acid, in comparison to placebo, in a study of patients mainly in remission [38]. However, the three other studies found significant improvements in DAS28, compared to placebo, from quercetin [39] (no significant effects on individual components), resveratrol [41] (effect on swollen and tender joints, ESR and CRP) and ubiquinone [40] (effects on swollen and tender joint) in patients with intermediate to high disease activity. The studies were considered to be of high [38] and intermediate quality [39,40,41]. The strength of evidence that alpha lipolic acid does not reduce disease activity in RA was considered moderate. The quality of evidence for the positive effects on DAS28 in RA was considered moderate for quercetin and ubiquinone, and low for resveratrol.

### 3.7. Studies on Pre-, Pro- and Synbiotics

Five studies investigated the effects of pre-, [42] pro- [43,44,45] and synbiotics [46] on disease activity in RA. All but two studies were conducted in Iran and two studies were from the same research group [44,46].

Alavi et al. compared a prebiotic supplement providing 1.3 g/day of fiber (aloe vera gel extract, arabinogalactan, gum ghatti, gum tragacanth, glucosamine) to control (rice flour) in 78 women and men with RA with intermediate disease activity. After six months, DAS28 was significantly worsened in the intervention group, compared to control. However, this difference was due to an improvement in the control group. The study quality was considered low [42].

Alipour et al. compared one daily capsule of L. casei 01 (10^8^ colony forming units) and maltrodextrin to maltrodextrin alone, among 60 women with RA. At baseline, the mean DAS28 indicated remission. After two months, the DAS28-CRP was lower in the intervention group compared to the control group [43] (effects on all individual components of DAS28). Study quality was considered intermediate.

In 2016, Zamani et al. compared a placebo (starch) to probiotic capsules containing L. casei (2 × 10^9^ colony forming units), L. Acidophilus (2 × 10^9^ colony forming units) and B. Bifidum (2 × 10^9^ colony forming units). The study was conducted among 60 women and men for two months. The baseline disease activity was intermediate. After two months, the probiotic intervention reduced DAS 28 in comparison to placebo (effects on CRP) [44]. The study quality was considered high.

Pineda et al. compared two daily capsules of probiotics containing 2 × 10^9^ of L. Rhamnous and L. Reuteri, in addition to inactive ingredients, to placebo (same ingredients but without the bacteria). The study included 29 patients, both men and women, and the disease activity at baseline was intermediate. After three months, there was no significant difference in disease activity between intervention and control [45]. The quality of the study was intermediate.

In 2017, Zamani et al. compared a synbiotic supplement containing the same probiotics as their previous trial in addition to 800 mg of inulin. The control group received placebo (starch). The study included 54 women and men, and the baseline disease activity was intermediate. After two months, disease activity was significantly reduced in the synbiotic group, in comparison to control (effects on CRP) [46]. The study quality was considered high.

Prebiotic fiber supplementation does not seem to reduce DAS28 in RA (low quality evidence). The strength of evidence was considered low that probiotic supplementation, with and without the addition of prebiotics, reduces DAS28 in RA. The evidence that probiotics containing L. casei improve disease activity compared to control was considered moderate.

## 4. Discussion

This systematic review included 33 RCT studies of diet or dietary supplements that reduce the disease activity of RA. The evidence was considered moderate that a Mediterranean style diet, ginger powder, cinnamon, saffron, quercetin, ubiquinone and probiotics containing *L. casei* reduce DAS28 in RA. The evidence was considered moderate that cranberry juice, folic acid and alpha lipoic acid do not reduce DAS28 in RA. The evidence for other interventions was very low or low, with only indicative effects. Most findings have never been verified in subsequent trials, making the strength of evidence limited. Consequently, there is a great need to confirm previous positive findings in order to conclude weather diet or dietary supplements can be used as adjuvant treatments to reduce disease activity in RA.

Dietary components can affect disease activity in RA through several direct or indirect interactions with the immune system. Long chain n-3 fatty acids found in fish can decrease the production of inflammatory eicosanoids, adhesion molecules and cytokines [47]. Antioxidants can reduce oxidative stress and thereby possibly symptoms and inflammation in RA [48]. In addition, dietary fiber, prebiotics or probiotics could have beneficial effects on disease activity in RA through modification of the microbiota [49]. Specific nutrients and alterations to microbiota can also affect the intestinal permeability, which may affect the immune response. Lastly, weight loss from dietary intervention could be beneficial for patients with RA, as obesity is associated with the more rapid progression of disabilities [50], but energy deficiency could also put patients at risk of malnutrition [51].

The findings from the current study indicate some positive effects of the Mediterranean diet that combines several of these factors, such as n3-fatty acids, fibre and dietary antioxidants [11]. The null effects from most of the n-3 fatty acid supplement interventions included in the current systematic review suggest that supplementation may be ineffective, despite doses of n-3 fatty acids unattainable through diet alone. The supplemented fatty acids will only contribute with a very small proportion of the total daily fat intake. The effect of manipulating the total fat quality of the diet is unclear and warrants further investigation. The findings of this review also show potentially beneficial effects from the supplements containing single antioxidants and spices with high antioxidant capacity. Antioxidant supplementation has been associated with both beneficial and adverse effects in many health conditions [52,53,54], and the safety of long-term high dose intake is uncertain. The doses of vitamin D given in the included studies, though ineffective in reducing disease activity, exceed the tolerable upper intake limit set by the European Food Safety Authority [55]. The doses given of spices in the included studies—approximately one teaspoon/day—might be achievable through diet alone. It is however noteworthy that this dose approximately corresponds to the tolerable daily intake for consumption of cinnamon, to avoid a toxic intake of coumarin [56]. Overall, high dose supplementation with nutrients, spices or antioxidants should be initiated with caution as adverse effects are possible.

Most of the included studies had study durations of two to four months. This seems sufficient to see changes in DAS28, according to the findings. Heterogeneity was observed in regards to which component of DAS28 was affected, as positive effects seemed to be mediated through different factors (inflammation, joint status or self-reported health) in different studies. All studies also included mostly or exclusively female participants, likely due to the overrepresentation of RA among women. However, most of the other aspects of study design, such as baseline disease activity, pharmacological treatment, disease duration, sample size and intervention, varied substantially between studies. These considerable heterogeneities in study design makes it difficult to summarize the results. This was expected, and therefore no meta-analysis was attempted.

A 2009 Cochrane review concluded that the effects of dietary intervention in RA are uncertain due to insufficient evidence [15]. Our conclusions for only DAS28 are similar, thus the evidence has not improved in the last 11 years despite new publications. This is in part due to a lack of significant results in recent dietary intervention trials, but also due to a lack of new dietary intervention studies. A previous systematic review and meta-analysis found no effects on DAS28 of n3-fatty acid supplementation [57], in line with our findings. However, other effects of n3-fatty acid supplementation were observed in the previous review, such as reduced inflammatory cytokines or improved blood lipid profile. Previous systematic reviews have found some indications of positive effects from diet on RA, other than on DAS28. These effects include the positive effects on pain and physical function of the Mediterranean diet [58], and on pain and improvement rates of fasting followed by vegetarian diet [59], but no evidence for effects of n-fatty acids or Mediterranean diet on fatigue [60]. The effects of diet on subjective outcomes such as pain and self-reported physical function are not unexpected from interventions that are difficult to blind, and where placebo effects may contribute significantly. Therefore, the relevance of these findings is unclear.

There was moderate strength of evidence for positive effects of probiotics containing L. casei, with or without prebiotics. This contradicts a 2017 meta-analysis that found no effect of probiotics on DAS [61], but with only partially the same studies included. The probiotics used in the studies included in the current systematic review comprised of different combinations of bacteria, but L. casei was used in most. As the results from the studies that used dietary probiotics did not show significant improvements in DAS28 [21,22], supplemental doses may be required. In addition, the bacteria strains in the supplemental studies were different from those in the diet interventions.

### Limitations and Strengths

A limitation of this work is the lack of outcomes other than DAS28. Other outcomes were excluded in order to make a comprehensible summary of results, while still enabling the inclusion of a broad range of interventions. DAS28 was chosen as the outcome as it is the most relevant assessment of disease activity in RA, and is used in clinical praxis. Still, including studies with other indicators of disease activity would have yielded a few more studies, but also would have complicated the interpretation of the results. Including other indicators of disease activity would also have meant the inclusion of mainly older studies. Choosing DAS28 as the outcome ensures results from current, relevant populations that have access to modern pharmacological treatment. Another possible limitation is that we only considered between-group differences in DAS28. However, DAS28 varies over time, and in studies that utilize a minimum threshold for inclusion, regression to the mean is to be expected. Therefore, within-group effects can be due to the natural fluctuations, rather than true effects. Other limitations are associated with the lack of high-quality studies, such as the lack of ITT analysis in the included papers, which means that the effects from single studies are likely inflated. The power was likely insufficient to detect significant effects in some of the studies, though most based their sample size on a priori power calculations. As few experiments have been reproduced, there is also a risk of publication bias where non-significant findings have not been reported. An indication of publication bias is the 13 conference abstracts identified in the searches that never seemed to have resulted in a published paper.

The strengths of this work include the use of two blinded assessors for article selection, quality assessment and data extraction. When one assessor had co-authored an included paper, a third blinded assessor performed quality assessment to ensure a fair appraisal. In addition, a skilled librarian performed all searches in three databases, safeguarding high quality searches that would include all relevant papers.

## 5. Conclusions

Based on the available results from RCT studies, evidence for the effects of specific diets, food items, spices, micronutrients, antioxidants and pre-, pro- and synbiotics on DAS28 is limited. Positive findings need confirmation in future high-quality studies.

## Figures and Tables

**Table 1:** Randomized controlled studies on effects of diet, food and spices on Disease Activity Score in Rheumatoid Arthritis.

Author, Year, Country [Ref]	Participants	Population	Study Duration	Intervention	Control	DAS28 Main Results	Adjusted Analysis	Study Quality	Comments
Sköldstam 2012 Sweden [20] RCT UB	N randomized: 56 N control: 27 N intervention: 29 Total drop out: 9%	80% women 49% MTX use Disease activity: Intermediate	12 weeks	Mediterranean diet Foods provided: Some meals in the initial 3 weeks. Margarine, olive oil, canola oil, frozen vegetables, tea.	Usual diet Foods provided: Some meals in the initial 3 weeks	DAS28 ESR I: 4.4 → 3.9 C: 4.3 → 4.3 *P* = 0.047	Unadjusted	B	No relevant power calculation Weight loss in intervention group Study violations not excluded
Nenonen 1998Finland [21] RCT UB	N randomized: 43 N control: 21N intervention: 22 Total drop out: 5%	86% women 35% MTX use Disease activity: Intermediate	3 months	Uncooked vegan diet Foods provided: All foods	Usual omnivore diet Foods provided: None	DAS28: I: 3.26 → 3.01 C: 3.44 → 3.46 *P* = 0.7	Adjusted for weight change	C	No relevant power calculation Weight loss in intervention group Calculation of DAS28 unclear
Vadell 2019Sweden [22] RCOT SB (assessors)	N randomized: 50 N control: N intervention: Total drop out: 12%	77% women 66% MTX use Disease activity: Intermediate	10 weeks	Anti-inflammatory, Mediterranean style diet, rich in fruit, berries, vegetable, nuts, whole grain, low fat dairy, fish and vegetable oil Foods provided: Breakfast, main meal, one snack per day for 5 d/w	Average Swedish diet, high in red meat, refined grains, butter, quark, protein bars Foods provided: Breakfast, main meal, one snack per day for 5 d/w	DAS28 ESR: I: 3.39 → 3.05 C: 3.42 → 3.27 *P* = NS	Adjusted for baseline, diet sequence, batch and starting diet	A	No drop out analysis but few dropouts Weight stable during both diets Adjusted for baseline and carry over effects
Thimoteo 2019Brazil [23] RCT UB	N randomized: 41N intervention: 23 N control:18 Total drop out: 7%	100% women 63% MTX use Disease activity: Intermediate	3 months	Usual diet plus 500 mL/d of reduced calorie cranberry juice	Usual diet	DAS28 ESR: I: 3.48 → 2.99 C: 3.59 → 3.52 *P* = NS	Unadjusted	B	No drop out analysis but few drop outs Excluded those with poor compliance
Lindqvist 2018Sweden [24] RCOT SB (assessors)	N randomized: 39 N control: 19 N intervention: 20 Total drop out: 41%	100% women 60% MTX use Disease activity: Intermediate	11 weeks	5 weekly meals with 75 g of blue mussels in addition to normal diet Foods provided: all 5 meals/w	5 weekly meals with meat/chicken in addition to normal diet Foods provided: all 5 meals/w	DAS28 (ITT) I: 3.96 → 3.40 C: 3.95 → 3.88 *P* = 0.023	Unadjusted	B	High drop out Did not consider carry over effects in analysis NS effects on DAS28 ESR in PP analysis DAS28 CRP also lower after intervention diet
Aryaeian 2019 Iran [25] RCT DB	N randomized: 70N intervention: 35 N control: 35 Total drop out: 10%	89% women 97% MTX use Disease activity: Intermediate	12 weeks	1500 mg ginger powder in 2 capsules daily	Similar capsules with wheat flour	DAS28 ESR: I: 4.73 → 3.44 C: 4.51 → 4.30 *P* = 0.003	Unadjusted	B	No relevant power calculation No drop out analysis but few drop outs
Shishehbor 2018 Iran [26] RCT DB	N randomized: 40 N intervention: 20 N control: 20 Total drop out: 10%	100% women 78% MTX use Disease activity: High	8 weeks	2000 mg cinnamon powder in 4 capsules daily	Similar capsules with starch	DAS28: I: 6.04 → 3.92 C: 5.35 → 5.64 *P* < 0.001	Adjusted for baseline and menopausal status	A	Almost worse by control
Hamidi 2020 Iran [27] RCT DB	N randomized: 66 N intervention: 33 N control: 33 Total drop out: 8%	100% women % MTX use not specified Disease activity: Intermediate	3 months	100 mg/d saffron in 1 tablet	Placebo (100 mg hydroxyl-propylmethyl cellulose in 1 tablet)	DAS28 CRP: I: 5.09 → 4.33 C: 4.92 → 5.19 *P* < 0.001	Unadjusted	B	Almost worse by control No drop out analysis but few drop outs Excluded uncompliant participants, though few Did not adjust for baseline, despite slight imbalance

RCT; randomized controlled trial, RCOT; randomized crossover trial, UB; unblinded, SB; single blind, DB; double blind, N; number, MTX; metrotrexate, ITT; Intention to treat.

**Table 2:** Randomized controlled studies of effects of single nutrients or antioxidants on Disease Activity Score in Rheumatoid Arthritis.

Author, Year, Country (Ref)	Participants	Population	Study Duration	Intervention	Control	DAS28 Main Results	Adjusted Analysis	Study Quality	Comments
Galarraga 2008UK (Scotland) [28] RCT DB	N randomized: 97N intervention: 49 N control: 48 Total drop out: 40%	71% women 77% DMARD use Disease activity: Intermediate	12 weeks	10 g cod liver oil in capsules containing: 1500 mg EPA, 700 mg DHA, 800 µg vit A, 5 µg vit D, 20 IE vit E	Placebo (air filled capsules)	DAS28 CRP: I: 4.5 → 4.3 C: 4.5 → 4.3 *P* = 0.976	Unadjusted	B	Study duration was 9 mo but after 12 w NSAID was reduced Amount of n3/d unclear No drop out analysis High drop out
Das Gupta 2009 Bangladesh [29] RCT UB	N randomized: 100N intervention: 50 N control: 50 Total drop out: 19%	% of women not specified % MTX use not specified Disease activity: High	12 weeks	75 mg/d indomethacin 3 g/d n3 FA in capsules	75 mg/d indomethacin	DAS28: I: 7.2 → 4.2 C: 7.3 → 4.8 *P* < 0.05	Unadjusted	B	No clear aim or hypothesis No clear description of participant characteristics Calculation of DAS28 unclear
Dawczynski 2018 Germany [30] RCOT DB	N randomized: 38N intervention: 38 N control: 38 Total drop out: 34%	84% women % MTX use not specified Disease activity: Intermediate	10 weeks	8 g micro algae, enriched in 60 g sausage, 8 g tomato spread, 30 g milk powder Amount of n3/d: 2.36 g	8 g sun flower oil, enriched in 60 g sausage, 8 g tomato spread, 30 g milk powder	DAS28: I: 4.5 → 3.88 C: 3.99 → 4.13 *P* = 0.085	Adjusted for sequence and baseline value	B	Excluded those with poor compliance Calculation of DAS28 unclear
Dawczynski 2009 Germany [31] RCOT UB	N randomized: 45N intervention: 45 N control: 45 Total drop out: 13%	96% women % MTX use not specified Disease activity: Intermediate	12 weeks	40 g fat in the form of 200 g yoghurt, 30 g cheese and butter. Milk fat was in part exchanged with oils high in EPA, DHA and alpha linoleic acid. Amount of n3/d: 2.4 g	Commercial dairy products with similar fat content	DAS28: I: 4.45 → 4.32 C: 4.18 → 4.24 *P* = NS	Adjusted for sequence and baseline value	B	Excluded those with poor compliance Adjusted for potential carry over effects in analysis Calculation of DAS28 unclear No relevant power calculation
Remans2004 Netherlands [32] RCT DB	N randomized: 66N intervention: 33 N control: 33 Total drop out: 17%	82% women 62% MTX use Disease activity: High	4 months	Liquid nutritional supplement containing PUFA (1400 mg EPA 211 mg DHA, 40 mg DPA, 16 mg ALA) and micronutrients	Placebo drink with the same taste, odor and color but with artificial sweetener	DAS28: I: 5.36 → 5.58 C: 5.14 → 5.35 *P* = NS	Unadjusted	B	Slight gain in body weight in intervention group Calculation of DAS28 unclear No drop out analysis
Buondonno 2017 Italy [33] RCT DB	N randomized: 39 N intervention: 21 N control: 18 Total drop out: 8%	100% women 100% MTX use Disease activity: High	3 months	300 000 IU (7500 µg) of vitamin D3 administered once	Placebo	DAS28 ESR: I: ? → 5.6 C: ? →5.8 *P* = NS	Unadjusted	B	Unclear DAS28 at baseline No dropout analysis, but low drop out
Hansen 2014 USA [34] RCT DB	N randomized: 22 N intervention: 11 N control: 11 Total drop out: 0%	46% women % MTX use not specified Disease activity: Low	12 months	Month 1: 3*50 000 IU vitamin D2/week Month 2-11: 2*50 000 IU vitamin D2/month 1500 mg calcium daily	Placebo 1500 mg calcium daily	DAS28: I: 3.0 → 3.03 C: 2.54 → 2.96 *P* = NS	Unadjusted	C	Sparse methods and results No relevant power calculation Calculation of DAS28 not presented
Rastmanesh 2008 Iran [35] RCT DB	N randomized: 38N intervention: 18 N control: 18 Total drop out: 16%	100% women 100% DMARD use Disease activity: High	28 days	6000 mg of potassium in the form of enriched white grape juice	Placebo grape juice	DAS28: I: 5.86 → −0.69 C: 5.80 → −0.1 *P* < 0.01	Adjusted for baseline	B	Hypokalemic participants Short study duration Calculation of DAS28 unclear Compliance unclear Conflict of interest statement missing
Shishavan 2015 Iran [36] RCT DB	N randomized: 64N intervention: 32 N control: 32 Total drop out: 9%	100% women 91% MTX use Disease activity: Remission	8 weeks	10 µg/day of vitamin K1 as a chewable tablet	Placebo	DAS28 CRP: I: 1.74 → 1.59 C: 2.26 → 1.85 *P* = NS	Adjusted (baseline, duration, folic acid intake, energy intake and weight)	A	No drop out analysis, but few drop outs Participants in remission at baseline
Van Ede 2001 Netherlands [37] RCT DB	N randomized: 434N intervention 1: 143 N intervention 2: 147 N control: 144 Total drop out unclear	71% women 100% MTX use Disease activity: Intermediate	12 months	Intervention 1: 1 mg/day of folic acid (oral, intake in morning) Intervention 2: 2.5 mg/week of folinic acid (oral, within 24 h of MTX intake)	Placebo	DAS28 ESR: I 1: 4.8 → −1.5 I 2: 4.6 → −1.4 C: 4.7 → −1.5 P1 = NS P2 = NS	Unadjusted?	B	Drop out unclear Unclear if analysis is adjusted
Gargari 2015 Iran [38] RCT DB	N randomized: 70N intervention: 35 N control: 35 Total drop out: 7%	100% women 88% MTX use Disease activity: Remission	8 weeks	Two capsules of 1200 mg alpha lipoic acid	Placebo (1200 mg maltodextrin)	DAS28: I: 2.11 → 1.86 C: 2.14 → 1.98 *P* = 0.442	Adjusted for baseline value	A	Excluded those with poor compliance, but few No dropout analysis, but few dropouts Participants in remission at baseline Calculation of DAS28 unclear
Javadi 2016 Iran [39] RCT DB	N randomized: 50N intervention: 25 N control: 25 Total drop out: 20%	100% women 92% MTX use Disease activity: Low–intermediate	8 weeks	One capsule containing 500 mg quercetin	Placebo (lactose)	DAS28 ESR: I: 3.22 → 2.65 C: 3.13 → 3.11 *P* = 0.04	Adjusted for baseline value	B	Conflict of interest statement missing No drop out analysis Excluded those with poor compliance
Nachvak 2019 Iran [40] RCT DB	N randomized: 54N intervention: 27 N control: 27 Total drop out: 17%	89% women % MTX use not specified Disease activity: Intermediate	2 months	100 mg/day CoQ10 capsules	Placebo	DAS28 ESR: I: 5.01→2.34 C: 4.88→4.04 *P* < 0.001	Adjusted for baseline, age, sex, disease duration, medications, and total energy intake	B	Compliance unclear No drop out analysis Placebo unclear
Khojah 2018 Egypt [41] RCT UB	N randomized: 100N intervention: 50 N control: 50 Total drop out: 0%	68% women % MTX use not specified Disease activity: Intermediate	3 months	One capsule containing 1 g resveratrol daily	Regular treatment	DAS28 ESR: I: 4.62 → 3.12 C: 4.91 → 4.78 *P* < 0.001	Unadjusted	B	No placebo No relevant power analysis Compliance unclear

RCT; randomized controlled trial, RCOT; randomized crossover trial, UB; unblinded, SB; single blind, DB; double blind, N; number, MTX; metrotrexate, ITT; Intention to treat.

**Table 3:** Randomized controlled studies of effects of pre-, pro- or synbiotics on Disease Activity Score in Rheumatoid Arthritis.

Author, Year, Country [Ref]	Participants	Population	Study Duration	Intervention	Control	DAS28 Main Results	Adjusted Analysis	Study Quality	Comments
Alavi 2011 UK [42] RCT DB	N randomized: 78N intervention: 33 N control: 36 Total drop out: 22%	81% women % MTX use not specified Disease activity: Intermediate	6 months	Dietary fiber supplement, (Ambrotose complex: aloe vera gel extract, arabinogalactan, gum ghatti, gum tragacanth, glucosamine) – 1,3 g/day	Placebo (rice flour)	DAS28: I: ~4.1 → 4.0 C: ~4.3 → 3.8 *P* = 0.009	Unadjusted	C	No drop out analysis, but ITT Baseline DAS28 and treatment unavailable Calculation of DAS28 unclear
Alipour 2014 Iran [43] RCT DB	N randomized: 60N intervention: 30 N control: 30 Total drop out: 23%	100% women 76% MTX use Disease activity: Remisson	8 weeks	One daily capsule of L. casei 01, min 10^8^ colony forming units (plus maltrodextrin)	Placebo (maltrodextrin)	DAS28 CRP I: 2.56 → 2.07 C: 2.31 → 2.23 *P* = 0.039	Adjusted (baseline, BMI change, anxiety and menopausal status)	B	Excluded those with poor compliance No drop out analysis Remission at baseline
Zamani 2016 Iran [44] RCT DB	N randomized: 60N intervention: 30 N control: 30 Total drop out: 0%	85% women 97% MTX use Disease activity: Intermediate	8 weeks	Probiotic capsules containing L. acidophilus (2 × 10^9^ CFU), L. casei (2 × 10^9^ CFU), B. bifidum (2 × 10^9^ CFU)	Placebo (starch)	DAS28: I: 4.0 → 3.7 C: 4.1 → 4.0 *P* = 0.01	Adjusted for baseline, age, BMI	A	Calculation of DAS28 unclear
De Los Angeles Pineda 2011 Canada [45] RCT DB	N randomized: 29N intervention: 15 N control: 14 Total drop out: 10%	93% women 76% MTX use Disease activity: Intermediate	3 months	Two daily capsules of L. rhamnosus GR-1 and L. reuteri RC-14 (each 2 billion CFU), plus dextrose, potato starch, microcrystalline cellulose and magnesium stearate	Placebo (dextrose, potato starch, microcrystalline cellulose and magnesium stearate)	DAS I: 4.18 → ∆ − 2.1 C: 4.83 → ∆ − 2.9 *P* = 0.77	Unadjusted	B	No power calculationNo drop out analysis but few drop outs Compliance unclear
Zamani 2017 Iran [46] RCT DB	N randomized: 54N intervention: 27 N control: 27 Total drop out: 0%	85% women 96% MTX Disease activity: Intermediate	8 weeks	Synbiotic capsules containing L. acidophilus, L. casei, B. bifidum (each 2 × 10^9^ CFU), and 800 mg inulin	Placebo (starch)	DAS28: I: 4.2 → 2.6 C: 3.5 → 3.2 *P* < 0.001	Adjusted for baseline, BMI and age	A	Calculation of DAS28 unclear

RCT; randomized controlled trial, RCOT; randomized crossover trial, UB; unblinded, SB; single blind, DB; double blind, N; number, MTX; metrotrexate, ITT; Intention to treat, CFU; colony forming units, L; Lactobacillus, B; Bifidobacteria.

**Table 4:** Summary of quality of evidence for effects of diet, food and dietary supplements on Disease Activity Score in patients with Rheumatoid Arthritis.

Type of Intervention	Ref.	No Studies	Results for DAS28	Quality of Evidence	Comments
**Whole Diet Interventions**					
7Mediterranean style diet	[20,22]	2	Inconsistent, but suggestive	Moderate (+++)	Risk of bias: 0 Consistency: –1 Relevance: 0 Precision: 0 Publication bias: 0
Raw food diet	[21]	1	Non-significant compared to control	Very low (+)	Risk of bias: –2 Consistency: 0 Relevance: -1 Precision: 0 Publication bias: 0
**Single food items**					
Blue mussels	[24]	1	Improvement compared to control	Low (++)	Risk of bias: –1 Consistency: 0 Relevance: -1 Precision: 0 Publication bias: 0
Cranberry juice	[23]	1	Non-significant compared to control	Moderate (+++)	Risk of bias: –1 Consistency: 0 Relevance: 0 Precision: 0 Publication bias: 0
Ginger powder	[25]	1	Improvement compared to control	Moderate (+++)	Risk of bias: –1 Consistency: 0 Relevance: 0 Precision: 0 Publication bias: 0
Cinnamon powder	[26]	1	Improvement compared to control	Moderate (+++)	Risk of bias: 0 Consistency: 0 Relevance: 0 Precision: –1 Publication bias: 0
Saffron	[27]	1	Improvement compared to control	Moderate (+++)	Risk of bias: –1 Consistency: 0 Relevance: 0 Precision: 0 Publication bias: 0
**Nutrients**					
n-3 supplementation	[28,29,30,31,32]	5	Inconsistent, most studies show no effect	Low (++)	Risk of bias: –1 Consistency: –1 Relevance: 0 Precision: 0 Publication bias: 0
Vitamin D supplementation	[33,34]	2	Consistently no improvement compared to control	Low (++)	Risk of bias: –1 Consistency: 0 Relevance: 0 Precision: –1 Publication bias: 0
Vitamin K supplementation	[36]	1	No improvement compared to control	Low (++)	Risk of bias: 0 Consistency: 0 Relevance: –1 Precision: –1 Publication bias: 0
Folic acid supplementation	[37]	1	No improvement compared to control	Moderate (+++)	Risk of bias: –1 Consistency: 0 Relevance: 0 Precision: 0 Publication bias: 0
Potassium supplementation	[35]	1	Significant improvement compared to control	Low (++)	Risk of bias: –1 Consistency: 0 Relevance: –1 Precision: 0 Publication bias: 0
**Single antioxidants**					
Alpha lipoic acid	[38]	1	No improvement compared to control	Moderate (+++)	Risk of bias: 0 Consistency: 0 Relevance: –1 Precision: 0 Publication bias: 0
Quercetin	[39]	1	Improvement compared to control	Moderate (+++)	Risk of bias: –1 Consistency: 0 Relevance: 0 Precision: 0 Publication bias: 0
Resveratrol	[41]	1	Improvement compared to control	Low (++)	Risk of bias: –1 Consistency: 0 Relevance: 0 Precision: –1 Publication bias: 0
Ubiquinone (Q10)	[40]	1	Improvement compared to control	Moderate (+++)	Risk of bias: –1 Consistency: 0 Relevance: 0 Precision: 0 Publication bias: 0
**Pro-, pre- and synbiotics**					
Prebiotics	[42]	1	Significantly worse compared to control	Low (++)	Risk of bias: –2 Consistency: 0 Relevance: 0 Precision: 0 Publication bias: 0
Probiotics and synbiotics, overall Probiotics and synbiotics containing L Casei	[43,44,45,46] [43,44,46]	4 3	Inconsistent overall Consistent improvement compared to control	Low (+++) Moderate (+++)	Risk of bias: 0 Consistency: –2 Relevance: 0 Precision: 0 Publication bias: 0 Risk of bias: 0 Consistency: 0 Relevance: 0 Precision: 0 Publication bias: –1

## Supplementary Materials

The following are available online at https://www.mdpi.com/2072-6643/12/10/2991/s1, Table S1: Population, Intervention, Control, Outcome (PICO) statement, Table S2: Search strategy.
