# Supplementary material for: Do Interventions with Diet or Dietary Supplements Reduce the Disease Activity Score in Rheumatoid Arthritis? A Systematic Review of Randomized Controlled Trials
Source: Nutrients. 2020 Sep 29;12(10):2991. doi: 10.3390/nu12102991 (PMC7600426; doi:10.3390/nu12102991)
Supplement: Supplementary file 1 [file nutrients-12-02991-s001.pdf]

Table S1. PICO (Population, Intervention, Control, Outcome) statement

	**Inclusion criteria**	**Exclusion criteria**
**Participants**	Adults (>18 years) with Rheumatoid Arthritis	Other rheumatoid diseases, including Juvenile Rheumatoid Arthritis or Juvenile Idiopathic Arthritis
**Intervention**	Dietary intervention with either diet, foods or dietary supplements. This includes whole diet, specific foods, spices, nutrients, dietary antioxidants, pre-, pro- and synbiotics.	Natural remedies of a traditional medicinal type or herbal remedies
**Control**	Placebo, diet representing normal intake or habitual diet	Pharmacological treatment (not also given to intervention group) or high dose vs low dose
**Outcome**	Disease activity score 28 joint count (DAS28)	

Table S2. Search strategy

PubMed 20190624	Search string	Number of results
#16	#12 NOT #15	462
#15	#13 OR #14	
#14	animals[ti] OR animal[ti] OR rats[ti] OR rat[ti] OR mouse[ti] OR mice[ti] or monkey*[ti] or primate*[ti]	
#13	((animals[mh]) NOT (animals[mh] AND humans[mh]))	
#12	#10 AND #11	488
#11	"randomized controlled trial"[pt] OR "controlled clinical trial"[pt] OR randomized[tiab] OR randomised[tiab] OR placebo[tiab] OR "drug therapy"[sh] OR randomly[tiab] OR trial[tiab] OR groups[tiab] OR RCT[tiab]	
#10	#3 AND #6 AND #9	780
#9	#7 OR #8	275526
#8	DAS[tiab] OR DAS28[tiab] OR disease activity[tiab] OR patient acuity[tiab]	47092
#7	patient acuity[mesh]	237070
#6	#4 OR #5	3177270
#5	diet[tiab] OR dietary[tiab] OR food[tiab] OR nutrition*[tiab] OR probiotic*[tiab] OR prebiotic*[tiab] OR symbiotic*[tiab] OR supplement*[tiab] OR selenium[tiab] OR zinc[tiab] OR vitamin*[tiab] OR antioxidant*[tiab] OR curcuma*[tiab] OR tumeric[tiab] OR turmeric[tiab] OR ginger[tiab] OR zingiber[tiab] OR garlic[tiab] OR vegan*[tiab] OR vegetarian*[tiab] OR polyphenol*[tiab] OR spices[tiab] OR fatty acid*[tiab] OR omega*[tiab] OR plant[tiab] OR plants[tiab]	2132349
#4	diet, food, and nutrition[mesh] OR plants[mesh] OR nutrition therapy[mesh] OR fatty acids[mesh]	1989641
#3	#1 OR #2	141680
#2	rheumatoid arthritis[tiab] OR arthritis rheumatoid[tiab]	100672
#1	Arthritis, Rheumatoid[mesh]	108732

**Scopus 20190624**	**Search string** TITLE-ABS-KEY for #1-#6	**Number of results**
#8	#6 AND NOT #7	701
#7	TITLE(animals OR animal OR rats OR rat OR mouse OR mice OR monkey* OR primate*)	
#6	#4 AND #5	709
#5	randomized OR randomised OR placebo OR "drug therapy" OR randomly OR trial OR groups OR RCT	
#4	#1 AND #2 AND #3	1188
#3	DAS OR DAS28 OR “disease activity” OR “patient acuity”	526965
#2	diet OR dietary OR food OR nutrition* OR probiotic* OR prebiotic* OR symbiotic* OR supplement* OR selenium OR zinc OR vitamin* OR antioxidant* OR curcuma* OR tumeric OR turmeric OR ginger OR zingiber OR garlic OR vegan* OR vegetarian* OR polyphenol* OR spices OR fatty acid* OR omega* OR plant OR plants	4040696
#1	“rheumatoid arthritis” OR “arthritis rheumatoid”	187811

**Cochrane Library 20190624**	**Search string** TITLE-ABSTRACT-KEYWORDfor #1-#6	**Number of results**
#8	#6 AND NOT #7	376
#7	(animals OR animal OR rats OR rat OR mouse OR mice OR monkey* OR primate*):ti	
#6	#4 AND #5	376
#5	randomized OR randomised OR placebo OR "drug therapy" OR randomly OR trial OR groups OR RCT	
#4	#1 AND #2 AND #3	394
#3	DAS OR DAS28 OR “disease activity” OR “patient acuity”	15495
#2	diet OR dietary OR food OR nutrition* OR probiotic* OR prebiotic* OR symbiotic* OR supplement* OR selenium OR zinc OR vitamin* OR antioxidant* OR curcuma* OR tumeric OR turmeric OR ginger OR zingiber OR garlic OR vegan* OR vegetarian* OR polyphenol* OR spices OR fatty acid* OR omega* OR plant OR plants	182324
#1	“rheumatoid arthritis” OR “arthritis rheumatoid”	14827
